# Celiac Disease Presenting as Fever of Unknown Origin

**DOI:** 10.1155/2013/676327

**Published:** 2013-07-18

**Authors:** Megan J. Cooney, Wael El-Matary

**Affiliations:** ^1^Department of Pediatrics, AE405-840 Sherbrook Street, Winnipeg, MB, Canada R3A 1S1; ^2^Section of Pediatric Gastroenterology, Department of Pediatrics and Child Health, University of Manitoba, Winnipeg, MB, Canada

## Abstract

Celiac disease (CD) is a common autoimmune enteropathy that occurs, in affected individuals, with exposure to gluten in the diet and improves with removal of dietary gluten. Although CD is readily considered in patients with classical presentations of the disease, atypical manifestations may be the only presenting symptoms. We present a case of CD in a 16-year-old female presenting as fever of unknown origin, which has not been reported previously. The postulated mechanism for fever in CD and the importance of clinicians having a low threshold for considering CD in the differential diagnosis of fever of unknown origin and other enigmatic clinical presentations is discussed.

## 1. Introduction

Celiac disease (CD), or gluten sensitive enteropathy, is a chronic inflammatory autoimmune enteropathy [1]. Classically it presents between the ages of 6 and 24 months, after gluten is introduced into the diet, with signs and symptoms of malabsorption including diarrhea, failure to thrive, abdominal pain, and signs of nutrient and vitamin deficiency [1]. Although only decades ago CD was thought to be rare, it is now accepted that the worldwide prevalence is 0.5 to 1 percent [1]. Furthermore, epidemiological screening studies suggest that the prevalence of CD is 1 : 133 in patients without risk factors or symptoms [2]. Given that atypical manifestations may be the only presenting symptoms of this common disease it is imperative that clinicians consider CD when investigating patients with enigmatic clinical presentations [2]. We present a case of CD presenting as fever of unknown origin.

## 2. Case

A 16-year-old female presented with one month of intermittent fever, night sweats, rigors, malaise, a 5 kg weight loss, and migratory arthralgias. The fever was noncyclic, and she was well in between episodes. Nausea, emesis, diarrhea, constipation, and abdominal pain were absent. She had no sick contacts and no out of country travel.

Her father had arthritis of unknown etiology in his early twenties that subsequently resolved, and there was a paternal cousin with alopecia of unknown etiology. She was on no medications.

On physical examination, she appeared well, and her height and weight plotted between the 75–90th and 90–97th percentiles, respectively. There were no signs of anemia, and clubbing was absent. Her liver was 2 centimeters below the costal margin, and her spleen tip was palpable. 

Inflammatory markers were mildly elevated (erythrocyte sedimentation rate (ESR) = 42 mm/hr, C-reactive protein (CRP) = 17.8 mg/L). Hemoglobin (Hb) was within normal limits (12.2 g/dL), as were other cell lines. There was a normal immunoglobulin profile, complement components 3 and 4 were within normal limits, and rheumatoid factor was negative. Thyroid function tests demonstrated a mildly depressed thyroid-stimulating hormone (0.33 *μ*IU/mL) with normal free triiodothyronine (4.76 pmol/L) and thyroxine (15.5 pmol/L). Antistreptolysin O antibody titre was not elevated (<200 units/mL). Throat swab culture and blood cultures demonstrated no growth. Serological tests for human immunodeficiency virus-type 1, human immunodeficiency virus-type 2, hepatitis C virus, hepatitis A virus, Epstein-Barr virus, cytomegalovirus, herpes simplex virus, and West Nile virus were negative. The presence of hepatitis B surface antibody and the absence of hepatitis B surface antigen were consistent with immunization. Additionally, serological tests for *Toxoplasma gondii*, *Bartonella henselae*, *Borrelia burgdorferi*, *Coxiella burnetti*, and *Blastomyces dermatitidis *were negative. *Brucella* spp. antibody was reactive (1 : 40), but confirmatory testing revealed this to be a false positive. An abdominal ultrasound and an abdominal computed tomography (CT) scan demonstrated mild splenomegaly but were otherwise normal.

Of note was that she had a tissue transglutaminase (tTG) immunoglobulin G (IgG) that was 15 units (normal <20) and tTG immunoglobulin A (IgA) that was 17 units (normal <20), indicating a negative screen for CD. A tagged white blood cell scan performed in October 2011 was normal. 

Later that month, a 5-day episode of fever greater than 40 degrees Celsius associated with nausea and emesis prompted further investigation. Her inflammatory markers remained mildly elevated (ESR = 32 mm/hr, CRP = 23.9 ml/L). She had developed a microcytic anemia (Hb = 9.8 g/dL, mean corpuscular volume = 78.9 fL), while her other cell lines remained within normal limits. Anti-double-stranded deoxyribonucleic acid and antinuclear antibodies were negative. Her serum albumin was slightly low (3.4 g/dL). A barium meal and small bowel follow-through was normal. 

An upper GI endoscopy and colonoscopy were done to exclude inflammatory bowel disease. The colonoscopy was unremarkable, while the upper GI endoscopy revealed gastric inflammation but was otherwise normal. However, on microscopic pathology duodenal biopsies were consistent with celiac disease, demonstrating villous blunting associated with an increased chronic inflammatory infiltrate in the lamina propria and significant intraepithelial lymphocytosis (MARSH IIIb). Repeat celiac screen was positive with tTG IgA 23 units and endomysial antibody IgA positive (1 : 10). Subsequent HLA typing revealed the presence of HLA-DQ2. She was started on a gluten-free diet (GFD), and at last followup was afebrile with normalized albumin and inflammatory markers.

## 3. Discussion

To our knowledge, there are no published reports of celiac disease presenting as fever of unknown origin. Leonardi et al. described the case of 3-year-old child with CD who presented with recurrent febrile infections and moderate neutropenia [3]. Following the introduction of a GFD, the patient's neutrophil count normalized, and the febrile infections remitted. Our patient exhibited neither neutropenia nor diagnosed infections during her episodes of pyrexia.

Fever results from cell production and release of cytokines with endogenous pyrogenic activity. Cytokines that are known endogenous pyrogens include interleukin (IL)-1, IL-6, tumor necrosis factor (TNF)-*α*, and the interferons (INF) [4]. They act in the anterior hypothalamus, inducing the synthesis of prostaglandins which raise the hypothalamic set point for temperature.

In CD, the activation of gluten specific T cells in the gastrointestinal mucosa induces production of proinflammatory cytokines, including TNF-*α* and INF-*γ*. These cytokines induce intraepithelial lymphocytosis, resulting in epithelial damage and many common clinical manifestations of CD [5]. This immune mediated production of endogenous pyrogenic cytokines is the postulated mechanism for fever in CD. 

In 2007, the Canadian Celiac Association Survey published a statistical analysis reporting that atypical symptoms are the presenting features in more than 25 percent of patients with biopsy-confirmed CD [6]. To decrease diagnostic latency it is imperative to expand the definition of what is considered a symptom of CD and view CD with the same propensity for enigmatic presentation as other autoimmune diseases. Using our case as an example, if CD was more readily considered in the investigation of fever of unknown origin, more cases may be promptly diagnosed. Early consideration of CD and screening of patients may decrease the severe complications associated with delaying commencement of a GFD [7]. Clinicians need to have a low threshold for considering CD in their differential diagnoses, especially in the case of fever of unknown origin. 
